# Prolapsus of the Rectum in Children

**Published:** 1865-02

**Authors:** 


*Prolapsus of the Rectum in Children.*—With adults the excision of some of the anal folds or of a portion of the prolapsed mucous membrane may be of use; but M. Guersant, of Paris, has found that the actnal cautery lightly applied round the anus was very successful. He burns four points close to the orifice in a square, one just by the coccyx, one opposite that process in front, and two others on either side. The action should reach as far as the sphincter; simple cold-water dressing is employed afterwards, and some of the little patients have recovered in a few days.

